# Gene Therapy Using Adeno‐Associated Virus Serotype 8 Encoding TNAP‐D_10_ Improves the Skeletal and Dentoalveolar Phenotypes in *Alpl* ^−/−^ Mice

**DOI:** 10.1002/jbmr.4382

**Published:** 2021-06-15

**Authors:** Yuka Kinoshita, Fatma F Mohamed, Flavia Amadeu de Oliveira, Sonoko Narisawa, Koichi Miyake, Brian L Foster, José Luis Millán

**Affiliations:** ^1^ Sanford Children's Health Research Center Sanford Burnham Prebys Medical Discovery Institute La Jolla CA USA; ^2^ Division of Biosciences College of Dentistry, The Ohio State University Columbus OH USA; ^3^ Department of Biochemistry and Molecular Biology Nippon Medical School Tokyo Japan

**Keywords:** GENETIC ANIMAL MODELS, OSTEOMALACIA AND RICKETS, DENTAL BIOLOGY, BONE HISTOMORPHOMETRY, BONE μCT

## Abstract

Hypophosphatasia (HPP) is caused by loss‐of‐function mutations in the *ALPL* gene that encodes tissue‐nonspecific alkaline phosphatase (TNAP), whose deficiency results in the accumulation of extracellular inorganic pyrophosphate (PP_i_), a potent mineralization inhibitor. Skeletal and dental hypomineralization characterizes HPP, with disease severity varying from life‐threatening perinatal or infantile forms to milder forms that manifest in adulthood or only affect the dentition. Enzyme replacement therapy (ERT) using mineral‐targeted recombinant TNAP (Strensiq/asfotase alfa) markedly improves the life span, skeletal phenotype, motor function, and quality of life of patients with HPP, though limitations of ERT include frequent injections due to a short elimination half‐life of 2.28 days and injection site reactions. We tested the efficacy of a single intramuscular administration of adeno‐associated virus 8 (AAV8) encoding TNAP‐D_10_ to increase the life span and improve the skeletal and dentoalveolar phenotypes in TNAP knockout (*Alpl* ^−/−^) mice, a murine model for severe infantile HPP. *Alpl* ^−/−^ mice received 3 × 10^11^ vector genomes/body of AAV8‐TNAP‐D_10_ within 5 days postnatal (dpn). AAV8‐TNAP‐D_10_ elevated serum ALP activity and suppressed plasma PP_i_. Treatment extended life span of *Alpl* ^−/−^ mice, and no ectopic calcifications were observed in the kidneys, aorta, coronary arteries, or brain in the 70 dpn observational window. Treated *Alpl* ^−/−^ mice did not show signs of rickets, including bowing of long bones, enlargement of epiphyses, or fractures. Bone microstructure of treated *Alpl* ^−/−^ mice was similar to wild type, with a few persistent small cortical and trabecular defects. Histology showed no measurable osteoid accumulation but reduced bone volume fraction in treated *Alpl* ^−/−^ mice versus controls. Treated *Alpl* ^*−/−*^ mice featured normal molar and incisor dentoalveolar tissues, with the exceptions of slightly reduced molar enamel and alveolar bone density. Histology showed the presence of cementum and normal periodontal ligament attachment. These results support gene therapy as a promising alternative to ERT for the treatment of HPP. © 2021 The Authors. *Journal of Bone and Mineral Research* published by Wiley Periodicals LLC on behalf of American Society for Bone and Mineral Research (ASBMR).

## Introduction

Hypophosphatasia (HPP) is an inborn error‐of‐metabolism caused by loss‐of‐function mutations in the *ALPL* gene, which encodes tissue‐nonspecific alkaline phosphatase (TNAP).^(^ ^1^, ^2^, ^3^ ^)^ TNAP is expressed in bones, teeth, liver, and kidney; its deficiency leads to mineralization defects caused by the accumulation of extracellular inorganic pyrophosphate (PP_i_), one of the major substrates of TNAP and a potent inhibitor of hydroxyapatite crystal formation and propagation.^(^ ^4^ ^)^ Murine studies have demonstrated that mineralizing skeletal and dental cells, including osteoblasts, chondrocytes, ameloblasts, odontoblasts, and cementoblasts, express TNAP and thus would be affected in HPP.^(^ ^5^, ^6^ ^)^

HPP patients suffer from distinctive rickets and/or osteomalacia with a broad range of severity, as well as dental defects. There are seven major forms of HPP: life‐threatening perinatal and infantile (OMIM#241500), benign perinatal, mild, and severe childhood (OMIM#241510), adult (OMIM#136300), and odonto‐HPP (OMIM#146300).^(^ ^7^ ^)^ Patients with perinatal HPP, the gravest form of HPP, often die in utero or soon after birth because of severe skeletal hypomineralization, respiratory failure due to thoracic cage dysplasia and hypoplastic lungs, and elevated intracranial pressure due to craniosynostosis.^(^ ^8^, ^9^, ^10^, ^11^ ^)^ Dentoalveolar phenotypes, including premature exfoliation of primary teeth, periodontal disease, and enamel alternations, are commonly observed in patients with all forms of HPP.^(^ ^12^, ^13^, ^14^ ^)^

There are more than 400 mutant alleles identified for the *ALPL* gene (the *ALPL* mutation database http://alplmutationdatabase.hypophosphatasie.com), and their genotype/phenotype correlations are not well understood.^(^ ^15^, ^16^ ^)^ The inheritance pattern of perinatal and infantile HPP is often autosomal recessive, with most patients being compound heterozygotes for pathogenic *ALPL* mutations that result in almost null alkaline phosphatase (ALP) activity, but some are homozygous for recessive alleles and most adult and odonto‐HPP patients harbor a single dominant‐negative *ALPL* allele.^(^ ^17^, ^18^ ^)^

Asfotase alfa is a recombinant fusion protein comprising the TNAP ectodomain, a human IgG1 Fc domain for one‐step purification, and a terminal deca‐aspartate (D_10_) motif for mineral targeting. In a murine model for infantile HPP, TNAP knockout (*Akp2* ^*−/−*^ or *Alpl* ^*−/−*^) mice, treatment with daily subcutaneous injections of asfotase alfa preserved life span, improved skeletal phenotypes, and prevented epileptic seizures and dental defects.^(^ ^19^, ^20^, ^21^ ^)^ In humans, subcutaneous injections of asfotase alfa, three to seven times a week, in children or adults with HPP has demonstrated substantial and sustained efficacy with a good safety profile. Asfotase alfa saved lives of severe neonatal and infantile HPP and improved bone mineralization, motor function, and quality of life in adult HPP.^(^ ^8^, ^22^, ^23^, ^24^, ^25^, ^26^ ^)^ At the same time, the patient burden of multiple injections per week to maintain the efficacy of asfotase alfa and the associated medical cost^(^ ^27^, ^28^ ^)^ have prompted preclinical studies of alternative strategies for treating HPP.

A human chimeric recombinant alkaline phosphatase, ChimAP, and several forms of virus vectors expressing TNAP‐D_10_ have shown to prolong life, prevent seizures, and improve the skeletal phenotype of *Alpl* ^*−/−*^ mice.^(^ ^29^, ^30^, ^31^, ^32^, ^33^ ^)^ A single intravenous injection of a lentiviral or adeno‐associated virus type 8 (AAV8) vector encoding TNAP‐D_10_ led to sustained correction of the skeletal phenotype of *Alpl* ^*−/−*^ mice, but the consequential wide distribution of vector genome to the whole body raised concern about possible transduction into germ cells.^(^ ^30^ ^)^

In this study, we treated *Alpl* ^*−/−*^ mice with a single intramuscular injection of AAV8 vector encoding TNAP‐D_10_ and performed a detailed analysis of bone microstructure, dentoalveolar phenotype, inorganic PP_i_ metabolism, and ectopic calcification to assess the efficacy and safety of viral vector‐mediated gene therapy.

## Materials and Methods

### Mouse model of infantile HPP

TNAP knockout (*Alpl* ^*−/−*^) mice were created by inserting a Neo cassette into exon 6 of the mouse *Alpl* gene via homologous recombination.^(^ ^34^ ^)^ *Alpl* ^−/−^ mice phenocopy human infantile HPP, showing normal appearance and being indistinguishable from other siblings at birth.^(^ ^35^ ^)^ They display almost zero circulatory ALP activity, develop epileptic seizures, become cachectic, and die by 10 to 12 days postnatal (dpn) without additional supportive treatment.^(^ ^35^ ^)^ *Alpl* ^−/−^ mice were maintained in a 12.5% C57Bl/6 and 87.5% 129 J background and genotyped by PCR using genomic DNA extracted from toe samples within 5 days after birth. All animals (breeders, nursing mothers, pups, and weanlings) in this study were given free access to regular diet (2018 Teklad global 18% protein extruded rodent diets or 2019 Teklad global 19% protein rodent diets, Envigo, Indianapolis, IN, USA) with a standard level of vitamin B6, increased level of which was reported to improve the life span of *Alpl* ^−/−^ mice.^(^ ^19^, ^34^, ^35^ ^)^ The institutional Animal Care and Use Committee (IACUC) approved all the animal studies.

### Virus vector encoding the human TNAP‐D_10_ cDNA

TNAP‐D_10_ contains recombinant human soluble TNAP (sALP) and a deca‐aspartate (D_10_) sequence at the C terminus, which enables TNAP to target mineralized tissues, such as bone and teeth.^(^ ^19^ ^)^ The human IgG1 Fc domain present in asfotase alfa to enable a one‐step purification is absent from the product of this vector as purification is not required for viral‐vector mediated in vivo expression. Recombinant AAV type 8 vector encoding TNAP‐D_10_ (AAV8‐TNAP‐D_10_, aka ARU‐2801) was generated using the HEK293 cell line by the triple transfection method, purified, and then titrated as previously reported.^(^ ^36^, ^37^, ^38^ ^)^ Recombinant AAV type 8 vector encoding GFP (AAV8‐GFP) was used as a control to evaluate viral vector‐related adverse effects.^(^ ^39^ ^)^ Eleven *Alpl* ^−/−^ mice (male *n* = 6, female *n* = 5) and 14 wild‐type (WT) controls (male *n* = 7, female *n* = 7) were included in this study. After genotyping, all the *Alpl* ^−/−^ mice received a single injection of AAV8‐TNAP‐D_10_ at a dose of 3 × 10^11^ vector genomes (vg)/body into the quadriceps femoris within 5 dpn. Three control WT mice received the same dose of AAV8‐TNAP‐D_10_ to assess its effect on PP_i_ metabolism and soft‐tissue calcification when endogeneous TNAP activity is present. Seven WT mice received the same amount of control AAV8‐GFP vector and 4 WT mice were untreated; their data were combined and analyzed together as WT because of the lack of substantial differences between them. Mice were euthanized at 70 dpn by exsanguination, after intraperitoneal administration of Avertin. In mice, 70 dpn is the end of adolescence, equivalent to a human age of 20 years,^(^ ^40^ ^)^ and root formation and cellular cementum formation have been completed.^(^ ^20^, ^29^, ^41^ ^)^ For the analysis of untreated *Alpl* ^−/−^, 17 WT, 7 heterozygote (*Alpl* ^+/−^), and 10 *Alpl* ^−/−^ pups were collected at 10 dpn and euthanized by exsanguination after intraperitoneal administration of Avertin.

### Sample collection and biochemical analyses

Body weight of the mice was measured at 35 and 70 dpn. Blood was collected from the orbital sinus of isoflurane‐anesthetized mice using Pasteur pipets every 4 weeks after injection. Spot urine samples were collected simultaneously.

Blood was collected into two types of BD Microtainers coated with either clot activator or lithium heparin (Becton, Dickinson and Company, Franklin Lakes, NJ, USA). Blood for plasma collection and urine samples were placed on ice. Microtainers were then spun at 7000*g* for 10 minutes. Twenty microliters of heparin‐plasma were deproteinized using a Microcon‐10 kDa Centrifugal Filter Unit with Ultracel‐10 membrane (MilliporeSigma, Merck KGaA, Darmstadt, Germany), centrifuged at 14,000*g* for 20 minutes. Urine was diluted 1:3 with 10 mM HEPES. Samples were stored at −80°C for further analyses.

Plasma PP_i_ concentration was measured according to the protocol described previously.^(^ ^42^ ^)^ Five microliters of deproteinized plasma sample and the PP_i_ standard ranging from 0.125 μM to 20 μM (sodium pyrophosphate decahydrate, Sigma‐Aldrich, St. Louis, MO, USA) were added to 45 μL of assay mixture containing 90 μM adenosine 5′ phosphosulfate sodium salt (APS) (Sigma‐Aldrich), 22.5 μM MgCl_2_, 11.25 mM HEPES with 0.9 U/mL recombinant yeast ATP‐sulfurylase/MET3 (R&D Systems, Inc., Minneapolis, MN, USA). The mixture was incubated at 37°C for 30 minutes and heat‐inactivated at 90°C for 10 minutes. Ten microliters of each sample were then transferred into a 96‐well white‐bottom plate and mixed with 50 μL of BacTiter‐Glo Microbial Cell Viability Assay (Promega Corporation, Madison, WI, USA). Luminescence was measured by FilterMax F5 Multimode Microplate Readers (Molecular Devices, LLC, San Jose, CA, USA).

Serum ALP activity was measured using an enzymatic assay. Five microliters of serum and recombinant human ALP standards (0.099 to 216.0 ng/mL) were mixed with 95 μL of 10 mM pNPP in diethanolamine (DEA) buffer (pH 9.8) containing 1.0 mM MgCl_2_ and 20 μM ZnCl_2_. The increase in A405 nm was measured using OptiMax Microplate Absorbance Reader (Molecular Devices) for 15 minutes. QuantiChrom Calcium Assay Kit, QuantiChrom Creatinine Assay Kit, and QuantiChrom Urea Assay Kit (BioAssay Systems, Hayward, CA, USA) were used to measure serum and urine calcium, urine creatinine, and serum urea concentrations, respectively. Stanbio Phosphorus Liqui‐UV (EKF Diagnostics‐Stanbio Laboratory, Boerne, TX, USA) was used to measure serum and urine phosphorus concentrations.

### Quantitative polymerase chain reaction (qPCR)

Total RNA was extracted from the kidney using RNAeasy Plus Kit (Qiagen LLC, Germantown, MD, USA), and reverse transcription was carried out using PrimeScript RT Master Mix (Takara Bio USA, Inc., Mountain View, CA, USA). Real‐time qPCR was performed in a 384‐well plate in an Applied Biosystems 7900HT Fast Real‐Time PCR system (Thermo Fisher Scientific, Waltham, MA, USA) using cDNA equivalent to 25 ng total RNA and DyNAmo Flash SYBR Green qPCR Kit (Thermo Fisher Scientific). The reaction was run for 40 cycles at an initial temperature of 95°C for 7 minutes and then at 95°C for 10 seconds followed by 60°C for 15 seconds. Ct values were determined by the software, and the amplification of the target gene was normalized to that of 18S ribosomal RNA (Rn18s). Sequences of the primer pairs used for PCR are as follows: *Alpl* (NM_007431.3) F‐CTGCCACTGCCTACTTGTGT and R‐GATGGATGTGACCTCATTGC; *Ank* (progressive ankylosis protein) (NM_020332.4) F‐CTGCTGCTACAGAGGCAGTG and R‐GACAAAACAGAGCGTCAGCGA; *Enpp1* (ectonucleotide pyrophosphatase phosphodiesterase 1) (NM_001308327.1) F‐TCACGCCACCGAGACTAAATA and R‐ TGCAGTAGGGTGTCATGAAGG; *Abcc6* (ATP binding cassette subfamily C member 6) (NM_018795.2) F‐CATCTTGCCAGGAATCAACACT and R‐ACCAGGGACAAGCACAGGTA; *Il6* (interleukin 6) (NM_031168.2) F‐CAAAGCCAGAGTCCTTCAGAGAG and R‐TTAGCCACTCCTTCTGTGACTCC; *Tnf* (tumor necrosis factor*‐*alpha) (NM_013693.3) F‐CAGCCTCTTCTCATTCCTGCT and R‐GCCATTTGGGAACTTCTCATC; *Rn18s* (NR_003278.3) F‐TTGATTAAGTCCCTGCCCTTTGT and R‐CGATCCGAGGGCCTCACTA.

### Radiography and micro‐computed tomography (μCT)

Radiographic images of entire skeletons and forelimbs, hindlimbs, and skulls were obtained with a Faxitron MX‐20 DC4 (Chicago, IL, USA), using energy of 20 kV. Lengths of the femur, tibia, humerus, and radius were measured using ImageJ (Rasband WS, ImageJ, National Institutes of Health, Bethesda, MD, USA, https://imagej.nih.gov/ij/, 1997–2018). Head measurements were performed using the following landmarks^(^ ^43^, ^44^ ^)^: nose length, the length from the rostal point of intersection of nasal bones to the caudal point of intersection of nasal bones; cranial length, the length from the rostal point of intersection of nasal bones to the median (midline) point of the posterior margin of the foramen magnum; cranial width, the length from the right joining of squamosal body to zygomatic process of squamous portion of temporal bone to the left counterpart.

After fixation in 4% paraformaldehyde/PBS solution, hemi‐mandibles and femurs were scanned in a μCT 50 scanner (Scanco Medical, Bassersdorf, Switzerland) at 70 kV, 76 μA, 0.5 Al filter, 900 ms integration time, and 6 or 10 μm voxel dimension for mandibles and femurs, respectively. Reconstructed images were calibrated to five known densities of hydroxyapatite and analyzed using AnalyzePro (version 1.0; AnalyzeDirect, Overland Park, KS). For femurs, trabecular and cortical bones were segmented at 350 and 650 mg HA/cm^3^, respectively. The trabecular bone was traced using 50 slices (total of 0.5 mm) proximal to the distal femur growth plate to quantify bone volume (BV), total volume (TV), bone volume fraction (Tb.BV/TV), trabecular number (Tb.N), thickness (Tb.Th), spacing (Tb.Sp), connective density (1/mm^3^), and mineral density (Tb.BMD). For the cortical bone, 50 slices of the mid‐femur of each bone were used to quantify cortical bone volume fraction (Ct.BV/TV), cortical thickness (Ct.Th), porosity, and mineral density (Ct.BMD).

The first mandibular molar and associated alveolar bone were quantitively analyzed as previously described.^(^ ^45^ ^)^ The alveolar bone region of interest (ROI) included the area between 240 μm mesial to the most mesial point of the first molar mesial root and 240 μm distal to the most distal point of the distal root. Enamel was segmented above 1600 mg HA/cm^3^, while dentin/cementum and alveolar bone were segmented at 550 to 1600 mg HA/cm^3^.

### Tissue collection and histological studies

Skeletal and soft tissues were fixed in 4% paraformaldehyde/PBS solution and processed for histological analyses. Undecalcified fixed bone samples were placed in 30% sucrose/PBS solution and then cryo‐embedded in Optimal Cutting Temperature (OCT) compound (Tissue‐Tek, Torrance, CA, USA) in hexane dry ice bath and were sectioned by the Kawamoto method.^(^ ^46^ ^)^ The tibial and femur bones were placed in 0.125 M EDTA/10% formalin (pH 7.3) solution for 7 days for decalcification and were then paraffin embedded. Soft organs were either paraffin embedded or embedded in an OCT in an ethanol dry ice bath. Hematoxylin and eosin (H&E), von Kossa, von Kossa/van Gieson, and Safranin O staining were performed according to standard methods. Tissue ALP activity was assayed by incubating the OCT‐embedded sections in freshly mixed substrate solution made of one volume of 0.2 mg of Naphthol AS‐MX phosphate disodium salt per mL of water and one volume of 1.2 mg FAST Violet B salt per mL of 0.2 M Tris–HCl (pH 8.9) at room temperature for 60 minutes, and counterstaining in methyl green solution.^(^ ^47^ ^)^ Slides were observed under IX81 Olympus Microscope (Olympus Corporation, Center Valley, PA, USA) or scanned by Aperio AT2 system (Leica Biosystems of Leica Microsystems Inc., Buffalo Grove, IL, USA). Bone volume fraction (BV/TV) was measured using ImageJ.

Left hemi‐mandibles were fixed in Bouin's solution overnight, decalcified in an acetic acid/formalin/sodium chloride solution, processed for paraffin embedding, and sectioned at 5 μm thickness in the coronal plane. Paraffin sections were stained with H&E to assess tooth and associated periodontium. Immunohistochemistry (IHC) procedures were performed as described previously.^(^ ^48^ ^)^ Primary antibodies included monoclonal rat anti‐human alkaline phosphatase IgG (TNAP) (R&D Systems)^(^ ^49^ ^)^; polyclonal rabbit anti‐mouse bone sialoprotein (BSP) IgG (Dr Renny Franceschi, University of Michigan, Ann Arbor, MI, USA)^(^ ^50^ ^)^; and polyclonal LF‐175 rabbit anti‐mouse osteopontin (OPN) IgG (Dr Larry Fisher, NIDCR/NIH, Bethesda, MD, USA).^(^ ^48^ ^)^ In situ hybridization was performed for mouse *Alpl* with RNAscope 2.5 HD Detection reagent kit‐RED assay (Advanced Cell Diagnostics, Newark, CA, USA) following the manufacturer's instructions as previously described.^(^ ^51^ ^)^ For acellular cementum/periodontal ligament (PDL) analysis, H&E‐stained images captured with the same acquisition parameters were segmented using the color map function (5 Ramps) in ImageJ. This method is to pseudo‐color the images to make differences between pixel values more apparent for improved tissue visualization. The values for acellular cementum and mantle dentin thickness represent the average of three linear measurements taken at 90 μm, 100 μm, and 110 μm from the cemento‐enamel junction (CEJ) using the ImageJ straight‐line function. Mantle dentin is the outer layer, less mineralized dentin adjacent to acellular cementum. For assessing cellularity, a region of 5.5 mm^2^ area was defined 100 μm apical to the CEJ for counting cells in the PDL space.

### Statistics

All the statistical analyses were performed using GraphPad Prism version 9.0.0 (GraphPad Software, San Diego, CA, USA). Data are expressed as mean ± standard deviation (SD) in charts. Statistical analysis was performed by one‐way analysis of variance (ANOVA) followed by Tukey's multiple comparison test to explore the differences among control WT, treated *Alpl* ^−/−^, and treated WT mice. Unpaired *t* test with Welch's correction was performed to test the difference between WT and treated *Alpl* ^−/−^ mice. Significance was determined by *p* < 0.05.

## Results

### Improved survival and correction of plasma PP_i_ in AAV8‐TNAP‐D_10_ ‐treated *Alpl* ^−/−^ mice

**Fig 1:**
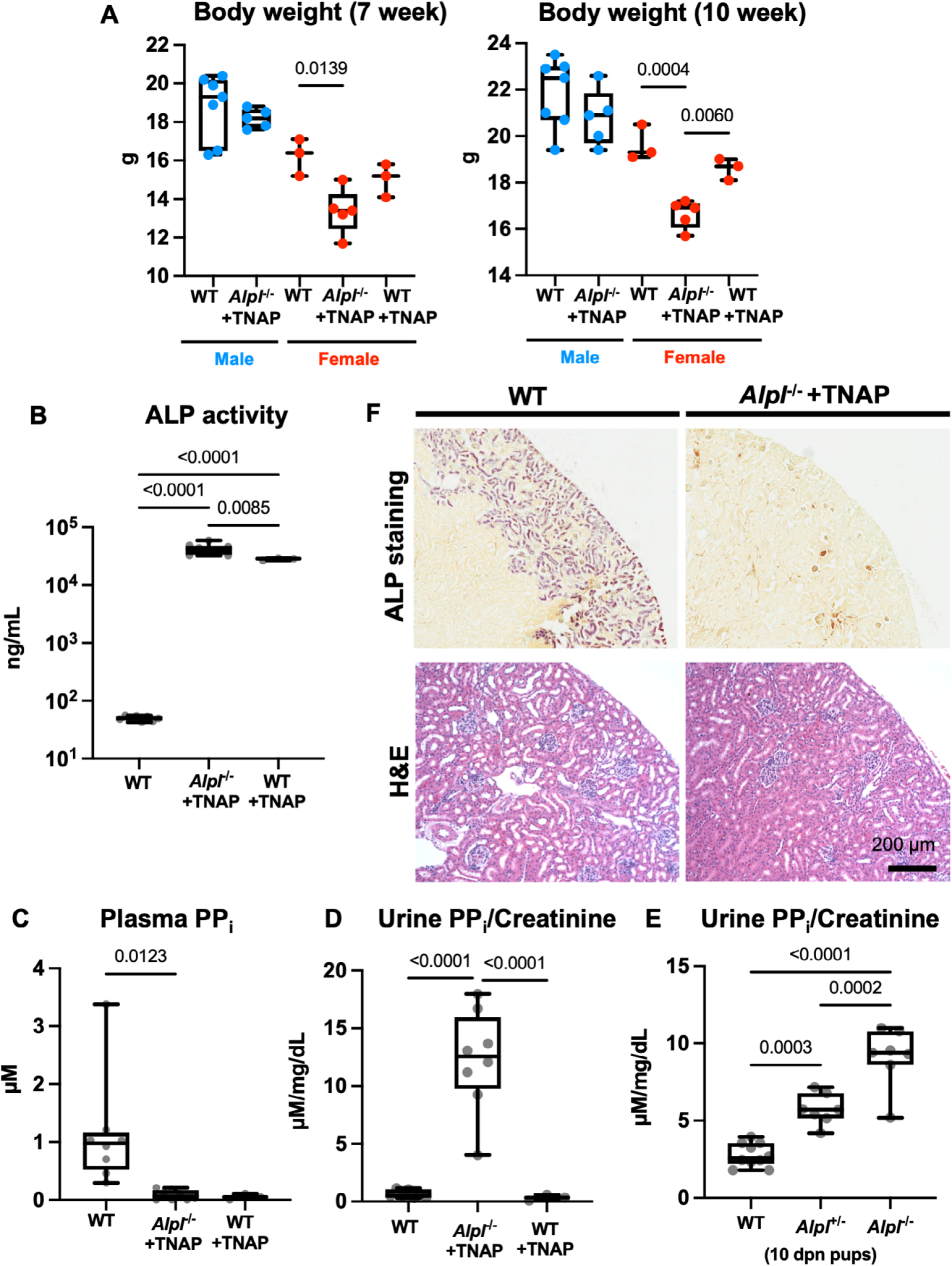
AAV8‐TNAP‐D_10_ improves survival and altered PP_i_ metabolism in AAV8‐TNAP‐D_10_‐treated *Alpl* ^−/−^ mice. (*A*) Average body weight of AAV8‐TNAP‐D_10_‐treated male *Alpl* ^−/−^ mice is comparable to WT littermates, although female *Alpl* ^−/−^ mice have lower body weight than WT. Blue and red circles in boxplots represent male and female data, respectively. (*B*) AAV8‐TNAP‐D_10_‐treated *Alpl* ^−/−^ and AAV8‐TNAP‐D_10_‐treated *Alpl* ^−/−^ WT mice show a significant increase in serum ALP activity compared with untreated WT mice. (*C*) Plasma PP_i_ levels of AAV8‐TNAP‐D_10_‐treated *Alpl* ^−/−^ mice are significantly lower than those of WT controls. (*D*) Urine PP_i_ concentrations of AAV8‐TNAP‐D_10_‐treated *Alpl* ^−/−^ mice remain significantly higher than both treated and untreated WT mice (70 dpn). (*E*) Urine PP_i_ concentrations of untreated *Alpl* ^−/−^ and heterozygote (*Alpl* ^*+/−*^) mice are higher than those of WT mice (10 dpn). (*F*) Histochemical staining of the kidney reveals no ALP activity in the proximal tubules of AAV8‐TNAP‐D_10_‐treated *Alpl* ^−/−^ mice (upper panel). Light microscopy does not reveal any apparent structural changes in the H&E‐stained glomeruli or renal tubules of AAV8‐TNAP‐D_10_‐treated *Alpl* ^−/−^ mice (lower panel). Statistical analysis was performed by unpaired *t* test with Welch's correction in male mice in (*A*) and one‐way ANOVA followed by Tukey's multiple comparison test in female mice in (*A*) as well as (*B*–*E*).

While untreated *Alpl* ^*−/−*^ pups die within 10 to12 days after birth, all *Alpl* ^−/−^ mice treated with AAV8‐TNAP‐D_10_ in this study did not develop epileptic seizures and were viable until the endpoint of the study at 70 dpn. The untreated *Alpl* ^−/−^ pups did not feed well and were significantly smaller than WT littermates at 10 dpn (Supplemental Fig. S1 *A*). In contrast, the *Alpl* ^−/−^ mice injected with AAV8‐TNAP‐D_10_ within 5 dpn showed catch‐up growth, and their body weight was not significantly different from WT littermates at 15 dpn (Supplemental Fig. S1 *B*). The body weight of AAV8‐TNAP‐D_10_‐treated male *Alpl* ^−/−^ mice were comparable to their WT littermates, but treated female *Alpl* ^−/−^ mice had lower body weight than their WT littermates both at 35 and 70 dpn (Fig. 1*A*).

Serum ALP activity was significantly higher in *Alpl* ^−/−^ and WT mice injected with AAV8‐TNAP‐D_10_, approximately 500 to 800 times higher than that of control WT mice (Fig. 1*B*; Table 1). There were no significant differences in serum calcium, serum phosphorus, serum urea, urine calcium, or urine phosphorus concentrations among experimental groups (Table 1). While plasma PP_i_ concentrations were almost undetectable in the *Alpl* ^−/−^ and WT mice treated with AAV8‐TNAP‐D_10_ (Fig. 1*C*; Table 1), urine PP_i_ concentrations of AAV8‐TNAP‐D_10_‐treated *Alpl* ^−/−^ mice remained significantly higher than WT mice at 70 dpn (Fig. 1*D*; Table 1). Elevated urine PP_i_ concentrations were also observed in untreated 10 dpn *Alpl* ^−/−^ mice and *Alpl* ^+/−^ mice (Fig. 1*E*). qPCR performed on RNA isolated from the kidney showed no expression of the *Alpl* gene in the *Alpl* ^−/−^ mice, and no significant differences were observed in other genes related to PP_i_ metabolism (*Ank, Enpp1*, and *Abcc6*), nor in genes related to inflammation (*Il6* and *Tnf*) (Table 2). Histochemical staining of the kidney and liver revealed no ALP activity in the renal proximal tubules and the branches of hepatic artery of AAV8‐TNAP‐D_10_‐treated *Alpl* ^−/−^ mice (Fig. 1*F*; Supplemental Fig. S2). Light microscopy did not reveal any apparent structural changes in the H&E‐stained glomeruli or renal tubules (Fig. 1*F*). No ectopic calcifications were observed in the aorta, coronary arteries, brain, or kidney by 70 dpn (Supplemental Fig. S3).

**Table 1:** Summary of Biochemical Analyses of 70 dpn Mice

Parameters	Genotype and treatment			ANOVA
	WT	*Alpl* ^−/−^ + TNAP	WT + TNAP	*p* Value
Serum ALP (ng/mL)	50.02 ± 4.51	42287.44 ± 8599.55^a^	27994.17 ± 1074.31^a^ ^,^ ^b^	<0.0001
Plasma PP_i_ (μM)	1.13 ± 0.90	0.08 ± 0.08^c^	0.05 ± 0.04	0.0094
Serum Ca (mg/dL)	10.06 ± 0.80	10.57 ± 0.83	10.37 ± 0.81	0.5342
Serum P (mg/dL)	4.33 ± 0.52	4.53 ± 0.87	3.52 ± 0.54	0.1740
Serum BUN (mg/dL)	53.77 ± 6.76	52.24 ± 8.00	48.31 ± 7.24	0.6144
Urine PP_i_/Cr (μM/mg/dL)	0.64 ± 0.31	12.24 ± 4.08^a^	0.33 ± 0.20^d^	<0.0001
Urine Ca/Cr	0.68 ± 0.46	0.51 ± 0.20	0.59 ± 0.41	0.7107
Urine P/Cr	1.30 ± 1.10	2.35 ± 3.21	2.56 ± 2.00	0.6855

WT = wild type; TNAP = tissue‐nonspecific alkaline phosphatase; ALP = alkaline phosphatase; PP_i_ = inorganic pyrophosphate; Ca = calcium; P = phosphorus; BUN = blood urea nitrogen; Cr = creatinine. Data are presented as mean ± standard deviation. Statistical analysis was performed by one‐way ANOVA followed by Tukey's multiple comparison test.

^a^ *p* < 0.0001 compared with WT.

^b^ *p* = 0.0085 compared with *Alpl* ^−/−^ + TNAP.

^c^ *p* = 0.0123 compared with WT.

^d^ *p* < 0.0001 compared with *Alpl* ^−/−^ + TNAP.

**Table 2:** Summary of qPCR Analysis of the Kidney

Genes	Genotype and treatment		Welch's *t* test
	WT	*Alpl* ^−/−^ + TNAP	*p* Value
*Alpl*	0.83 ± 0.25	0.04 ± 0.02	<0.0001
*Ank*	0.62 ± 0.21	0.94 ± 0.35	0.0608
*Enpp1*	0.95 ± 0.21	1.04 ± 0.23	0.4732
*Abcc6*	0.71 ± 0.13	0.72 ± 0.08	0.8705
*Il6*	0.39 ± 0.35	0.44 ± 0.32	0.8155
*Tnf*	1.16 ± 0.41	0.95 ± 0.33	0.3244

WT = wild type; TNAP = tissue‐nonspecific alkaline phosphatase. Delta‐delta Ct method was used to calculate relative gene expressions with *Rn18s* as a reference gene. Data are presented as mean ± standard deviation. Statistical analysis was performed by unpaired *t* test with Welch's correction.

### Improved bone microstructure in AAV8‐TNAP‐D_10_ ‐treated *Alpl* ^−/−^ mice

**Fig 2:**
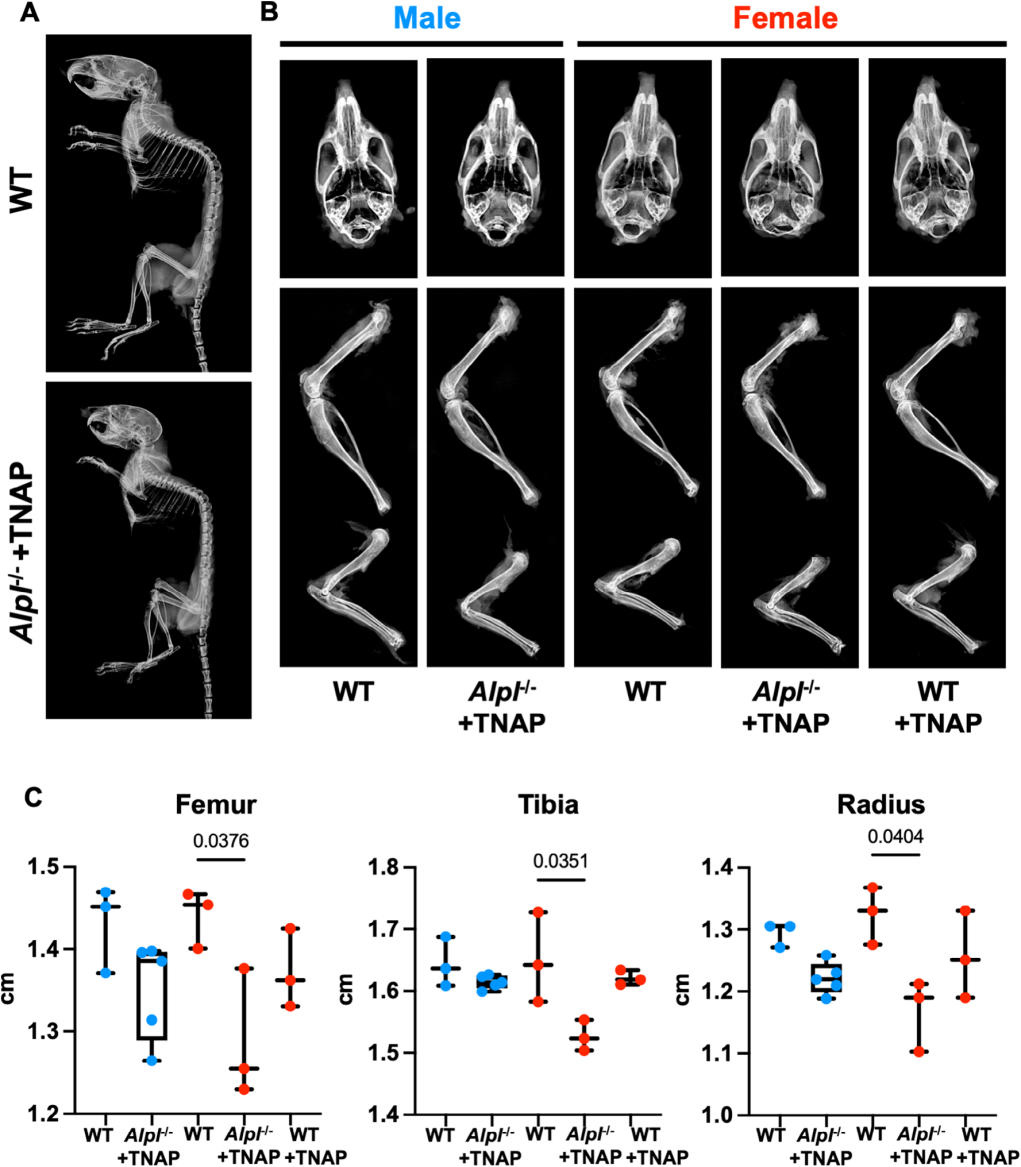
Improved radiographical findings in AAV8‐TNAP‐D_10_‐treated *Alpl* ^−/−^ mice. (*A*) Radiographs of the whole skeleton of a female WT littermate control and a female *Alpl* ^−/−^ mouse treated with AAV8‐TNAP‐D_10_. (*B*) AAV8‐TNAP‐D_10_‐treated *Alpl* ^−/−^ mice showed no signs of rickets, such as bowing and flaring of the metaphysis, and craniosynostosis. (*C*) AAV8‐TNAP‐D_10_‐treated female *Alpl* ^−/−^ mice have shorter limb lengths compared with their WT littermates. Statistical analysis was performed by unpaired *t* test with Welch's correction in male mice and one‐way ANOVA followed by Tukey's multiple comparison test in female mice. Blue and red circles in boxplots represent male and female data, respectively.

In previous studies, untreated 20 to 23 dpn *Alpl* ^−/−^ mice demonstrated profound skeletal abnormalities including reduced tissue mineral density and bone fractures.^(^ ^29^, ^52^ ^)^ Radiography of 70 dpn *Alpl* ^−/−^ mice treated with AAV8‐TNAP‐D_10_ revealed grossly normal skeletal development comparable to WT littermates (Fig. 2*A*). Treated *Alpl* ^−/−^ mice did not show features characteristic of rickets or osteomalacia, such as bowing of the long bones, enlargement of the ends of epiphyses, or fractures (Fig. 2*B*). Long bone lengths of treated male *Alpl* ^−/−^ mice were not significantly different from those of WT mice, while treated female *Alpl* ^−/−^ mice were significantly smaller than their WT littermates (Fig. 2*C*). Treated *Alpl* ^−/−^ mice did not show craniosynostosis (Fig. 2*B*). Nose length, cranial length, and cranial width were not significantly different among each treatment group in female mice, while the cranial lengths of AAV8‐TNAP‐D_10_‐treated *Alpl* ^−/−^ male mice were longer than those of WT male mice (Table 3).

**Table 3:** Summary of Head Measurements of 70 dpn Mice

Parameters (male)	Genotype and treatment			Welch's *t* test
	WT	*Alpl* ^−/−^ + TNAP	WT + TNAP	*p* Value
Nose length	0.96 ± 0.01	0.95 ± 0.06	N/A	0.9717
Cranial length	1.11 ± 0.02	1.23 ± 0.04	N/A	0.0033
Cranial width	1.05 ± 0.01	1.04 ± 0.02	N/A	0.7144

Parameters (female)	Genotype and treatment			ANOVA
	WT	*Alpl* ^−/−^ + TNAP	WT + TNAP	*p* Value
Nose length	1.00 ± 0.03	0.90 ± 0.11	0.97 ± 0.05	0.3211
Cranial length	1.15 ± 0.01	1.15 ± 0.07	1.15 ± 0.09	0.9952
Cranial width	1.03 ± 0.01	1.05 ± 0.03	1.06 ± 0.03	0.3647

WT = wild type; TNAP = tissue‐nonspecific alkaline phosphatase; N/A = no applicable data.

Data are presented as mean ± standard deviation. Statistical analysis was performed by unpaired *t* test with Welch's correction in male and one‐way ANOVA in female.

**Fig 3:**
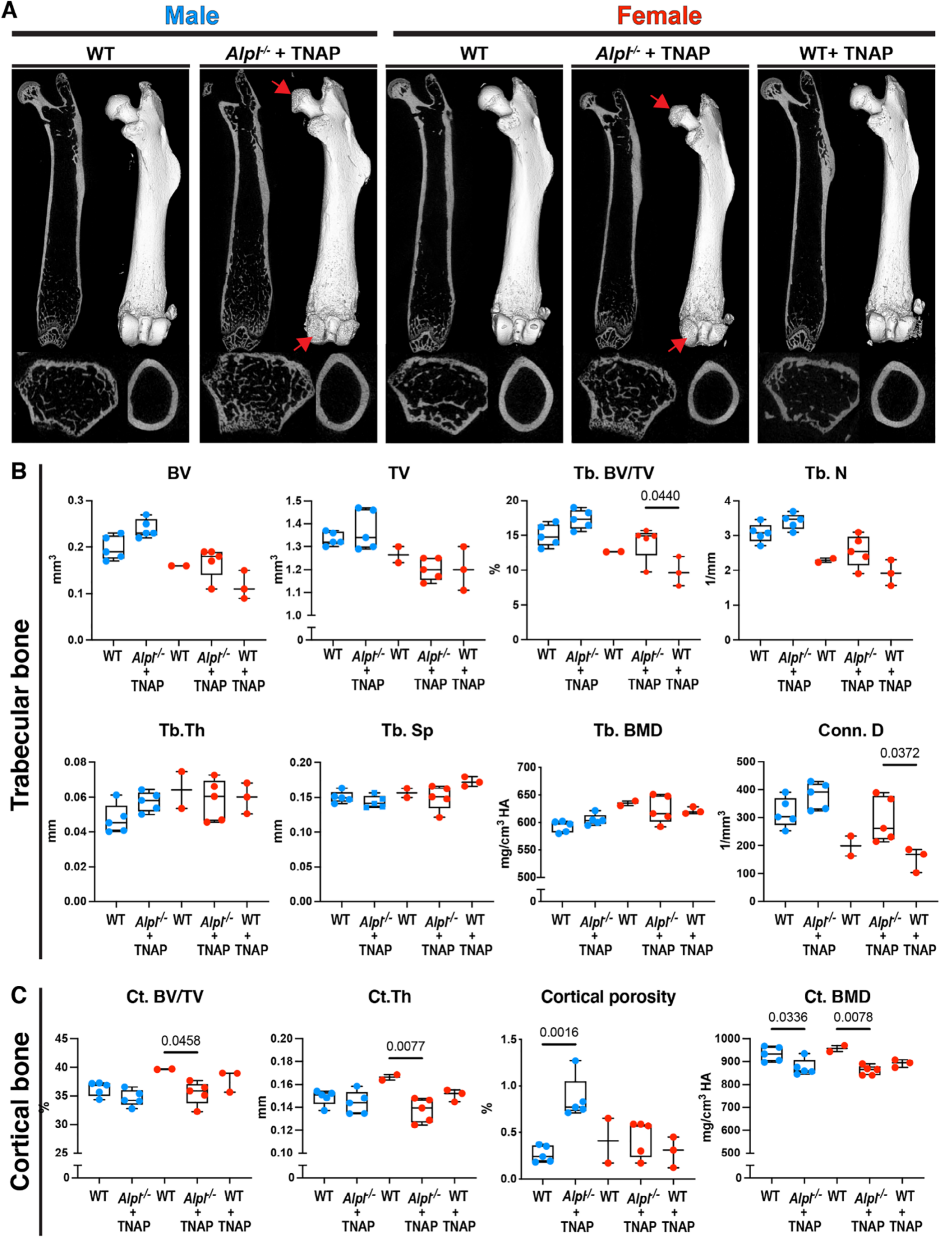
Partial normalization of bone microstructure in AAV8‐TNAP‐D_10_‐treated *Alpl* ^*−/−*^ mice. (*A*) 2D and 3D μCT images showing femurs from treated *Alpl* ^*−/−*^ mice compared with WT controls. Treated *Alpl* ^*−/−*^ females show shorter femurs compared with WT controls. Red arrows point to abnormal articular surfaces of medial and distal femurs in the treated *Alpl* ^*−/−*^ mice. (*B*) Quantification of trabecular bone parameters from 50 slices proximal to the growth plate of distal femurs. (*C*) Quantification of cortical bone parameters from 50 slices from femoral midshaft. Statistical analysis was performed by one‐way ANOVA followed by Tukey's multiple comparison test. Blue and red circles in boxplots represent male and female data, respectively.

Three‐dimensional μCT renderings of AAV8‐TNAP‐D_10_‐treated *Alpl* ^−/−^ femurs showed bone morphology similar to WT femurs; however, the bone was ~7% to 9% shorter in treated *Alpl* ^−/−^ females compared with WT controls (Figs. 2*C* and 3*A*). Notably, treated *Alpl* ^−/−^ mice had abnormal articular surfaces the femur joints compared with that of WT controls. Quantitative μCT analysis showed similar bone trabecular and cortical parameters in treated male and female *Alpl* ^−/−^ femurs compared with WT controls. Metaphyses of distal femurs showed no significant differences in TV, BV, or Tb.BV/TV among groups (Fig. 3*B*). However, treated *Alpl* ^*−/−*^ females showed increased Tb.BV/TV associated with improved trabecular connectivity compared with treated WT mice (Fig. 3*B*). No significant differences were detected in Tb.N, Tb.Th, Tb.Sp, or Tb.BMD among the groups. However, AAV8‐TNAP‐D_10−_treated *Alpl* ^−/−^ females showed reduced cortical BV/TV associated with decreased cortical thickness. Treated *Alpl* ^−/−^ males showed increased cortical porosity, but this did not significantly affect Ct.BV/TV. Also, AAV8‐TNAP‐D_10_ partially restored the Ct.BMD, both in males and females (Fig. 3*C*), indicating an incomplete rescue of cortical bone defects.

**Fig 4:**
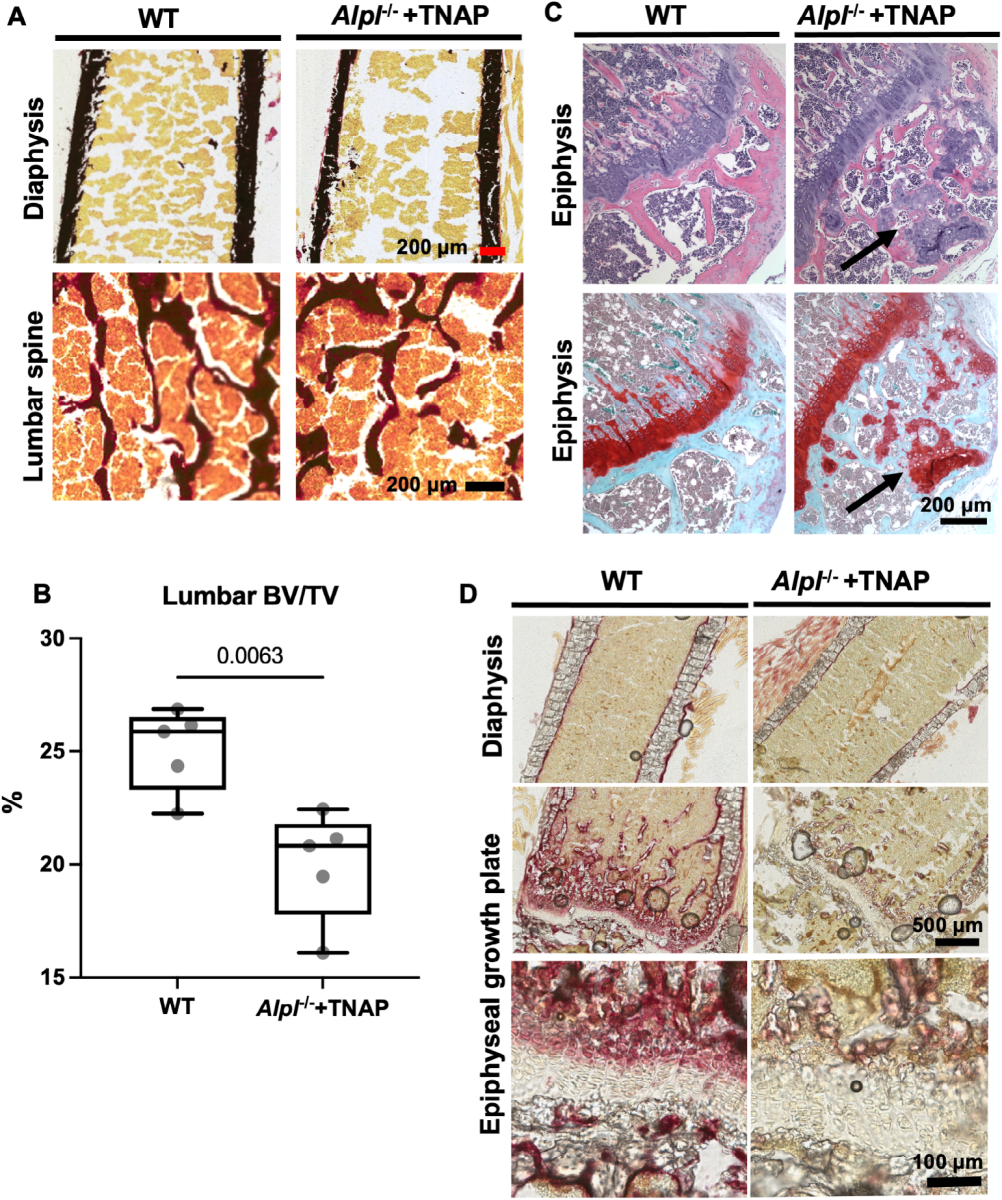
Improved histological findings in AAV8‐TNAP‐D_10_‐treated *Alpl* ^−/−^ mice. (*A*) Histological analysis of femur of WT and the AAV8‐TNAP‐D_10_‐treated *Alpl* ^−/−^ mice. Von Kossa/van Gieson staining of the femur bones and the lumbar spines show no measurable osteoid surface. (*B*) BV/TV values of the lumbar spine of AAV8‐TNAP‐D_10_‐treated *Alpl* ^−/−^ mice are significantly lower than that of WT mice. (WT *n* = 5; male *n* = 2, female *n* = 3; KO *n* = 3; male *n* = 1, female *n* = 2) Statistical analysis was performed by unpaired *t* test with Welch's correction. (*C*) H&E and Safranin O staining of the decalcified tibial bones show an abnormal distribution of chondrocytes in the secondary ossification center in AAV8‐TNAP‐D_10_‐treated *Alpl* ^−/−^ mice (black arrows). (*D*) Histochemical staining of the femur bones shows strong ALP activity in the hypertrophic zone of the epiphyseal growth plate, metaphysis, and diaphysis in WT mice, while only a subtle ALP activity is observed in the growth plate and diaphysis of AAV8‐TNAP‐D_10_‐treated *Alpl* ^−/−^ mice.

Von Kossa/van Gieson staining of femurs and lumbar spines showed no measurable osteoid surface (Fig. 4*A*), but the BV/TV values of lumbar spines of AAV8‐TNAP‐D_10_‐treated *Alpl* ^−/−^ mice were significantly lower than that of WT mice (Fig. 4*B*; WT *n* = 5; male *n* = 2, female *n* = 3, knockout [KO] *n* = 3; male *n* = 1, female *n* = 2). H&E and Safranin O staining of the decalcified tibias did not show the expansion of the tibial epiphyseal growth plate but showed an abnormal distribution of chondrocytes in secondary ossification centers in AAV8‐TNAP‐D_10_‐treated *Alpl* ^−/−^ mice (Fig. 4*C*; Supplemental Fig. S4). Histochemical staining of the femurs revealed strong ALP activity in the hypertrophic zone of the epiphyseal growth plate, metaphysis, and diaphysis of WT mice, while only subtle ALP activity was observed in the growth plate and diaphysis of AAV8‐TNAP‐D_10_‐treated *Alpl* ^−/−^ mice (Fig. 4*D*).

### AAV8‐TNAP‐D_10_ prevents HPP‐associated dentoalveolar defects in *Alpl* ^*−/−*^ mice

**Fig 5:**
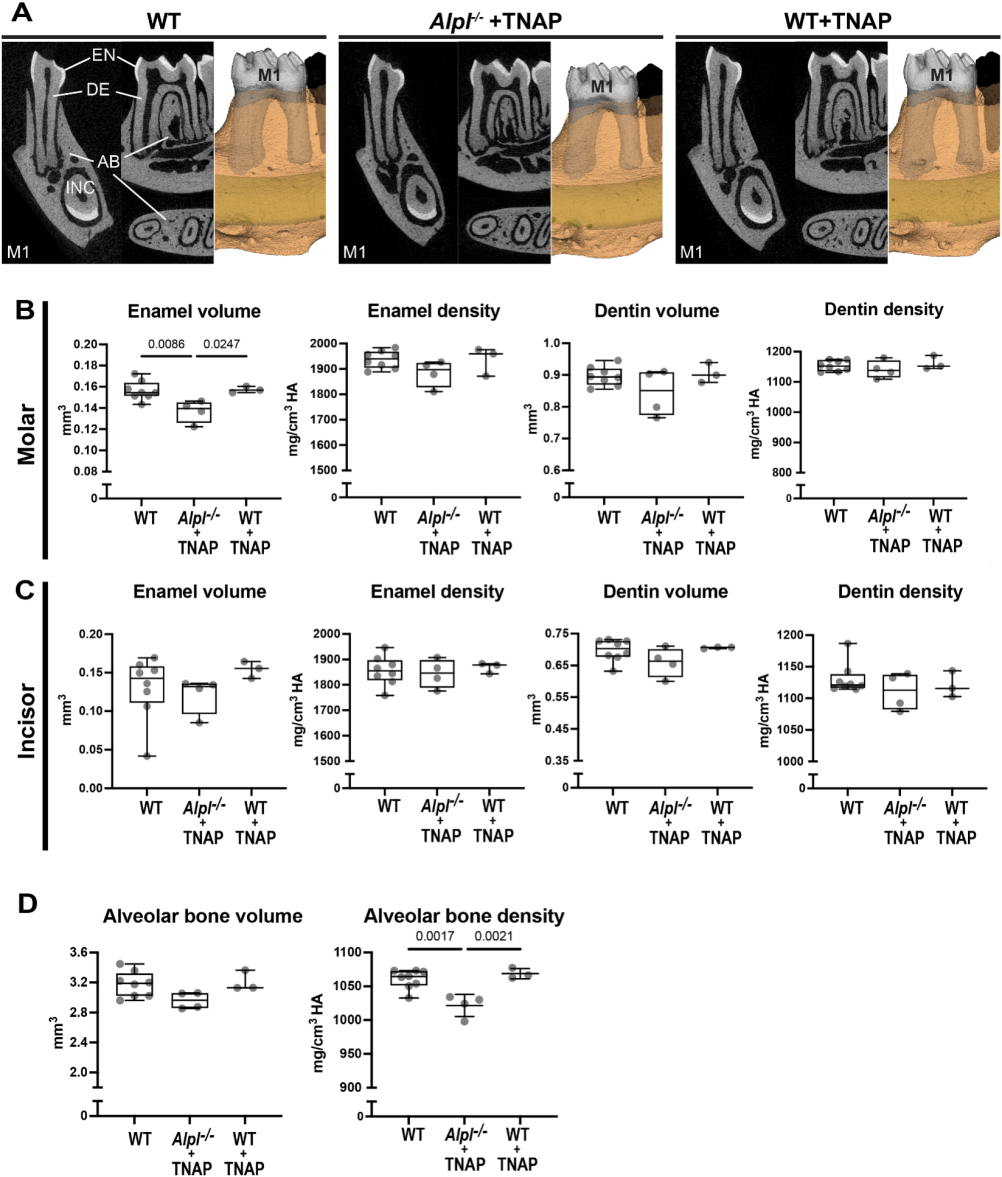
AAV8‐TNAP‐D_10_ prevents HPP‐associated dentoalveolar defects in *Alpl* ^*−/−*^ mice. (*A*) 3D and 2D μCT renderings of first molars (M1) and incisors (INC) exhibit normal tooth structures in AAV8‐TNAP‐D_10_‐treated *Alpl* ^*−/−*^ mice similar to those in WT controls (70 dpn). (*B*) First molars show no significant differences in enamel density, dentin volume, or dentin density among the groups. Tooth enamel shows decreased volume in treated *Alpl* ^*−/−*^ molars compared with WT controls. (*C*) Continually erupting incisor teeth show no significant defects in the volume or density of either enamel or dentin among the groups. (*D*) AAV8‐TNAP‐D_10_ significantly improves alveolar bone volume in treated *Alpl* ^*−/−*^ versus WT mice, but alveolar bone shows 4% less mineral density in treated *Alpl* ^*−/−*^ versus WT mice. Statistical analysis was performed by one‐way ANOVA followed by Tukey's multiple comparison test. EN = enamel; DE = dentin; AB = alveolar bone.

Data from male and female dentoalveolar tissues were combined because of lack of substantial sex‐related differences in previous reports of HPP mouse models^(^ ^20^, ^49^, ^53^ ^)^ and because we found no sex‐related trends in these data. Compared with untreated and treated WT controls, first mandibular molars and surrounding alveolar bone of AAV8‐TNAP‐D_10_‐treated *Alpl* ^−/−^ mice appeared grossly normal (Fig. 5*A*). Enamel volumes were 12% to 14% reduced in molars of AAV8‐TNAP‐D_10_‐treated *Alpl* ^−/−^ compared with WT groups, although no significant differences were observed in enamel mineral density (Fig. 5*B*). No differences in molar dentin volume or density were observed between AAV8‐TNAP‐D_10_‐treated *Alpl* ^−/−^ and WT groups (Fig. 5*B*). Continually erupting incisors of *Alpl* ^−/−^ mice, which harbor mineralization defects previously shown to be relatively resistant to treatment,^(^ ^20^, ^29^ ^)^ showed no differences in the volumes or densities of enamel or dentin compared with WT groups (Fig. 5*C*). Alveolar bone showed no differences in the volume between AAV8‐TNAP‐D_10_‐treated *Alpl* ^−/−^ mice and WT controls, although exhibited ~4% lower mineral density than WT groups (Fig. 5*D*).

**Fig 6:**
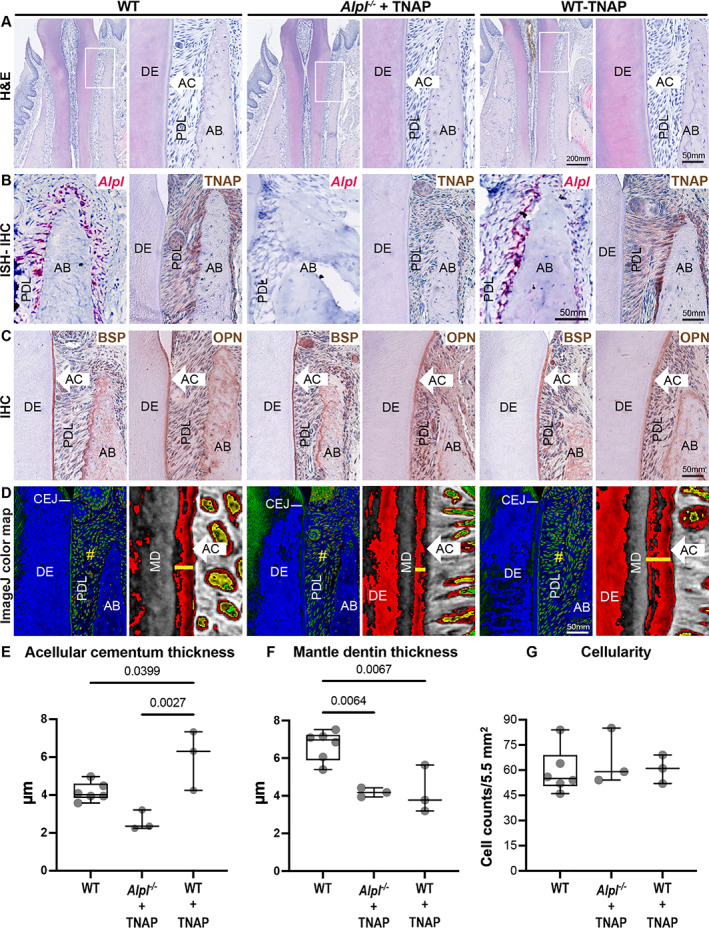
Improved cementum and PDL attachment in AAV8‐treated *Alpl* ^*−/−*^ mice. (*A*) H&E staining shows no evident dental defects in treated *Alpl* ^*−/−*^ mice compared with WT controls. Boxed regions are shown at higher magnification (left). (*B*) In situ hybridization with *Alpl* probe (left) confirms the absence of *Alpl* expression in treated *Alpl* ^*−/−*^ mice. TNAP IHC (right) shows weak staining (brown) around treated *Alpl* ^*−/−*^ alveolar bone (AB) and more robust staining in WT mice. (*C*) BSP IHC (left) shows evident staining (brown) in acellular cementum (AC, white arrow) and alveolar bone (AB) in treated *Alpl* ^*−/−*^ and WT mice. OPN IHC (right) shows comparable staining (brown) in AC and AB in treated *Alpl* ^*−/−*^ versus WT mice. (*D*) ImageJ color map (left) shows improved cellularity (yellow symbols) within the PDL space of treated *Alpl* ^*−/−*^ versus WT mice, and (right) shows the width of AC (yellow lines) in treated *Alpl* ^*−/−*^ versus WT mice. (*E*–*G*) Quantification of the thickness of acellular cementum, mantle dentin (MD), and cellularity in the PDL space. Statistical analysis was performed by one‐way ANOVA followed by Tukey's multiple comparison test.

Histology of AAV8‐TNAP‐D_10_‐treated *Alpl* ^−/−^ mouse mandibles revealed normal tooth structures largely indiscernible from those of WT controls, with similar morphology, tissue organization, presence of acellular cementum on root surfaces, and periodontal attachment (Fig. 6*A*). While in situ hybridization (ISH) showed no endogenous *Alpl* expression in AAV8‐TNAP‐D_10_‐treated *Alpl* ^−/−^ mouse tissues, immunohistochemistry (IHC) detected low‐level TNAP localized in the PDL (Fig. 6*B*), suggesting the contribution of AAV8‐mediated TNAP for amelioration of defects. AAV8‐TNAP‐D_10_‐treated *Alpl* ^−/−^ mice showed BSP and OPN immunostaining comparable to WT controls, confirming the identity of acellular cementum (Fig. 6*C*). Histomorphometry revealed no significant differences in acellular cementum thickness of treated *Alpl* ^−/−^ versus WT mouse molars, although TNAP‐D_10_‐treated WT teeth showed thickened acellular cementum (Fig. 6*D*, *E*). Outermost mantle dentin, which is enlarged and hypomineralized in humans with HPP and whose mineralization is delayed in *Alpl* ^*−/−*^ mice,^(^ ^45^ ^)^ was reduced in both treated *Alpl* ^*−/−*^ and WT mice (Fig. 6*D*, *F*). While untreated *Alpl* ^*−/−*^ mice showed PDL hypocellularity associated with defects in acellular cementum and PDL attachment (Supplemental Fig. S5), normal cellularity returned with treatment (Fig. 6*D*, *G*) and periodontal structures appeared intact.

## Discussion

We demonstrated in this study that a single, intramuscular injection of AAV8 vector encoding TNAP‐D_10_ during early neonatal days prolonged life span and markedly improved the skeletal and dentoalveolar phenotypes of the *Alpl* ^−/−^ mouse model of severe infantile HPP. Most findings from radiography, histology, and immunostaining showed near‐normal development of the limbs, skull, teeth, and associated periodontal tissues in treated *Alpl* ^−/−^ mice, while detailed analyses by μCT and histology revealed a remarkable improvement, yet slightly lower ratio in vertebral BV/TV and an incomplete rescue of cortical bone defects in the long bones of the AAV8‐TNAP‐D_10_‐treated *Alpl* ^−/−^ mice compared with WT mice. These corrections resulting from a single injection of vector‐mediated TNAP‐D_10_ are comparable to the skeletal and dental improvements observed in *Alpl* ^−/−^ mice under daily subcutaneous injection of asfotase alfa up to 52 days.^(^ ^19^ ^)^

### Efficacy of intramuscular delivery of AAV8‐TNAP‐D_10_

Previous approaches to correct HPP‐associated mineralization defects have met with mixed success or have had inherent limitations or drawbacks. ERT using asfotase alfa was shown to be very effective at correcting skeletal and dentoalveolar defects in *Alpl* ^−/−^ mice, with these translational studies leading to its approval in 2015 for treatment of patients with perinatal/infantile‐ and juvenile‐onset HPP, except in Japan, where asfotase alfa is approved for all ages.^(^ ^25^ ^)^ However, asfotase alfa ERT requires multiple injections per week, is associated with injection site reactions, and is expensive,^(^ ^27^, ^28^ ^)^ prompting additional preclinical studies of alternative strategies for HPP treatment. Daily subcutaneous injection of a soluble, non‐mineral‐targeting, recombinant chimeric alkaline phosphatase (ChimAP) prevented seizures, increased survival, but only partially improved the skeletal and dentoalveolar phenotypes in *Alpl* ^−/−^ mice.^(^ ^29^ ^)^ Studies using several different types of viral vectors with different modes of administration have been reported. Single intravenous injection of either lentiviral vector containing TNAP‐D_10_ (HIV‐TNAP‐D_10_) or AAV8‐TNAP‐D_10_ resulted in sustained elevation of circulatory TNAP and phenotypic correction in *Alpl* ^−/−^ mice, but the viral sequence was detected in soft tissues, including the liver, lung, and heart, raising a safety concern for oncogenicity of the integrated lentiviral vector in these tissues.^(^ ^30^, ^31^ ^)^ When compared with these previous methods, our approach described here using a single intramuscular injection of AAV8‐TNAP‐D_10_ combines a more practical gene therapy, potentially improved safety profile due to limited tissue distribution, and yet remains highly effective at correcting mineralization defects.

All forms of HPP include dental involvement, with premature tooth loss being one of the most common manifestations. Characteristic dentoalveolar abnormalities observed in untreated *Alpl* ^*−/−*^ mice include inhibition of tooth root acellular cementum formation, PDL detachment, and enamel and dentin mineralization defects.^(^ ^5^, ^20^, ^21^ ^)^ In our study, qualitative and quantitative analyses suggest that teeth and associated periodontal tissues significantly improved from AAV8‐TNAP‐D_10_‐mediated gene therapy. AAV8‐TNAP‐D_10_‐treated *Alpl* ^*−/−*^ mice showed normal formation and mineralization of molar and incisor enamel and dentin, and the restoration of acellular cementum, PDL cellularity, and PDL‐cementum attachment. Treated *Alpl* ^*−/−*^ mice also showed well‐developed alveolar bone with mildly reduced mineral density. An interesting finding is that AAV8‐TNAP‐D_10_ augmented acellular cementum in AAV8‐TNAP‐D_10_‐treated WT teeth, suggesting a potential effect on cementogenesis even in healthy mice. Circulating ALP levels were previously significantly correlated to mouse incisor acellular cementum thickness, supporting this mechanism.^(^ ^54^ ^)^ These results were comparable to those shown in the previous studies using daily subcutaneous injections of asfotase alfa.^(^ ^19^, ^20^, ^21^, ^52^ ^)^

### Insights into the distribution of TNAP‐D_10_ and the role of chondrocytes in HPP

In our study, histochemical staining of the femur showed significantly lower ALP activity in AAV8‐TNAP‐D_10_‐treated *Alpl* ^−/−^ mice in the epiphyseal growth plate and endosteum (Fig. 4*D*). Previous studies suggested that ALP activity observed in the bone of viral vector‐treated *Alpl* ^−/−^ mice decreases with age. Neonatal intravascular injection of AAV8‐TNAP‐D_10_ resulted in positive staining on the growth plate and the surface of the endosteal bone in 10 dpn mice.^(^ ^30^ ^)^ The tibias of mice that underwent transuterine intraperitoneal injections of AAV8‐TNAP‐D_10_ showed strong ALP activity in hypertrophic chondrocytes and on the surface of endosteal bones on day 14 but only scattered ALP signals in the cartilage zone on day 56.^(^ ^32^ ^)^ TNAP might not be actively incorporated into those sites at 70 dpn when skeletal maturity is achieved and avascularity of cartilage might limit accessibility of the therapeutic protein.^(^ ^55^ ^)^ Additionally, abnormal distribution of hypertrophic chondrocytes was observed in the tibial epiphysis in our AAV8‐TNAP‐D_10_‐treated *Alpl* ^*−/−*^ mice (Fig. 4*C*; Supplemental Fig. S4). The abnormal distribution of chondrocytes and irregularly arranged growth plates were previously reported in untreated 18‐ to 25‐dpn *Alpl* ^−/−^ mice and the *Alpl* ^−/−^ mice injected with a muscle‐directed vector, scAAV8‐TNAP‐D_10_.^(^ ^35^, ^56^ ^)^ In vitro experiments have shown that the inhibition of endogenous TNAP in hypertrophic chondrocytes impacts chondrocyte differentiation.^(^ ^57^ ^)^ Therefore, we hypothesize that the distribution of TNAP‐D_10_ may result in the normalization of growth plate irregularity and the remaining abnormal chondrocyte distribution in our treated *Alpl* ^−/−^ mice.

Lastly, three‐dimensional μCT showed abnormal articular surfaces of medial and distal femurs in the treated *Alpl* ^*−/−*^ mice (Fig. 3*A*). Articular surface disruption is often observed in the clinical syndrome of posttraumatic osteoarthritis, which occurs after the frequent injuries that cause chondral and subchondral bone damage.^(^ ^58^ ^)^ Joint complications such as arthropathies and periarticular calcification were reported in a mouse model of late‐onset HPP^(^ ^59^ ^)^ and adult patients with HPP.^(^ ^23^, ^60^ ^)^ Since the initial repair by chondrocytes is considered important in preventing the further progression of joint degeneration,^(^ ^58^ ^)^ the chondrocytes that lack TNAP activity might have caused an osteoarthritis‐like phenotype in our treated *Alpl* ^*−/−*^ mice. Further investigation is needed to clarify the etiology of joint degeneration in adult *Alpl* ^*−/−*^ mice.

### Considerations for AAV8‐TNAP‐D_10_ gene therapy

Therapies for HPP other than asfotase alfa have been tested in a clinical setting without rigorous preclinical and translational studies. In a cross‐sectional study that enrolled 51 patients with childhood and adult‐onset HPP, 2 of 4 patients who were treated with teriparatide (parathyroid hormone 1‐34) showed clinical and radiological improvement.^(^ ^61^ ^)^ In a phase IIA open‐label study targeting 8 adult patients with HPP, monoclonal antisclerostin antibody (BPS804) treatment resulted in increases in bone formation markers and bone mineral density.^(^ ^62^ ^)^ However, these anabolic agents have not been approved for HPP and an effective medical therapy for adult‐onset HPP patients is needed. At the same time, a novel therapy with fewer injections may further benefit patients with childhood‐onset HPP as well.

Concerns for AAV8‐TNAP‐D_10_ administration in human patients include continuously elevated circulatory ALP activity and its potential effects on PP_i_ metabolism and development or aggravation of ectopic calcification. In our studies, a single intramuscular injection of AAV8‐TNAP‐D_10_ resulted in extremely high circulatory ALP activity, more than 500 times higher than that of WT mice. Extracellular PP_i_ is known as a central regulator of biomineralization and is critical for controlling inappropriate soft‐tissue calcification in the body. Hydrolysis of PP_i_ by TNAP‐D_10_ may result in oversuppression of extracellular PP_i_ in soft tissues. However, AAV8‐TNAP‐D_10_‐treated *Alpl* ^−/−^ mice, with suppressed plasma PP_i_ concentrations, did not develop any soft‐tissue calcifications during our observational window of 70 dpn. Although it is unclear if the significant drop of PP_i_ concentrations in the treated animals to levels below the control group is due to in vivo metabolism of PP_i_ or ex vivo catabolism during the processing of the samples, this contrasts with previous results from genetically modified mouse models with targeted overexpression of TNAP in the vasculature. TNAP overexpression in the smooth muscle cells using *Tagln*‐Cre resulted in massive arterial calcifications in the ascending and descending aorta, carotid, and subclavian arteries.^(^ ^63^ ^)^ TNAP overexpression in endothelial cells using *Tie2*‐Cre resulted in the partial calcification in the arteries of the heart, kidney, mesentery, pancreas, and spleen.^(^ ^64^ ^)^ These mouse models showed 20 to 30 times higher circulatory ALP activity, while plasma PP_i_ concentrations were similar to those of WT. From these results, we conclude that neither circulatory ALP activities nor circulatory PP_i_ concentrations correlate with local PP_i_ concentrations, which cannot be measured in vivo but presumably are low in target tissues with high TNAP expression. Additionally, the elevated urine PP_i_ concentrations in TNAP‐D_10_‐treated *Alpl* ^−/−^ mice indicate that urine PP_i_ metabolism is mostly dependent on TNAP expressed in the luminal surface of kidney proximal tubules and independent of serum ALP activity and circulatory PP_i_ concentrations. The qPCR analysis of the kidneys of treated mice indicated that genes for primary regulators of systemic PP_i_ metabolism, including *Ank, Enpp1*, and *Abcc6*, were not affected by treatment.

In patients with HPP, with or without treatment, several types of ectopic calcifications are commonly observed: ocular calcification, nephrocalcinosis, and painful periarthritis.^(^ ^22^, ^23^, ^65^, ^66^, ^67^, ^68^, ^69^, ^70^, ^71^ ^)^ Our AAV8‐TNAP‐D10‐treated *Alpl* ^−/−^ mice did not develop nephrocalcinosis, kidney stones, or structural changes in the glomeruli or renal tubules as observed under light microscopy. qPCR did not show a significant increase in the inflammatory markers in the kidney. These mice did not develop hypercalcemia or hypercalciuria (Table 1). Therefore, we hypothesized that abnormal mineral metabolism, such as hypercalcemia and hypercalciuria observed in untreated HPP, might facilitate the development of nephrocalcinosis rather than an inflammatory response associated with TNAP deficiency.^(^ ^72^ ^)^ This study was terminated at 70 dpn; therefore, long‐term effects of AAV8‐TNAP‐D_10_ with the presence of age‐related ectopic calcifications remain unknown but are an area for further study. We have previously shown that asfotase alfa binds to the sites of ectopic calcification in the TNAP overexpression‐induced vascular calcification mouse models (*Tagln*‐Cre and *Tie2*‐Cre).^(^ ^73^ ^)^ Therefore, we cannot rule out the possibility that the continuous administration of TNAP‐D_10_, either subcutaneously or as a gene therapy, may lead to the development or aggravation of ectopic calcification in some HPP patients.

There are several limitations to our study. First, the skeletal growth of treated female *Alpl* ^−/−^ mice was not as well corrected as in male *Alpl* ^−/−^ mice, which suggests the possibility of sex differences in response to AAV8‐TNAP‐D_10_; however, there are no established sex differences among patients with HPP,^(^ ^3^ ^)^ and most previous in vivo studies did not indicate the sex differences in *Alpl* ^−/−^ mice. Therefore, further study is necessary to explore if sex affects phenotype and treatment response in HPP. Second, we did not assess the reproductive capacity of the mice treated with the virus vector and the health of their offspring in our study design. Finally, vector‐host interactions such as immune‐mediated responses and the emergence of neutralizing antibodies (NAb) against the virus capsid and/or transgene product are known as the major challenges that virus‐vector‐mediated gene therapy has to overcome before its clinical application.^(^ ^74^, ^75^, ^76^ ^)^ We did not perform a dose‐ranging study to determine the optimal dose of virus vector that can minimize the risk of immune responses and can correct the skeletal phenotype of the *Alpl* ^−/−^ mice. We did not analyze the presence of circulating NAb against AAV8 or TNAP‐D_10_ in our study. These limitations will be the basis for continuing studies.

In conclusion, a single intramuscular injection of AAV8‐TNAP‐D_10_ successfully corrected the skeletal and dentoalveolar phenotypes of otherwise lethal HPP mice without apparent complications in other tissues. Our results support gene therapy as a safe and effective approach and a potential alternative to ERT for the treatment of HPP.

## Disclosures

All authors state that they have no conflicts of interest.

### PEER REVIEW

The peer review history for this article is available at https://publons.com/publon/10.1002/jbmr.4382.

## Supporting information


**Supplemental Fig. S1.** (*A*) Untreated *Alpl* ^−/−^ mice were significantly smaller than WT mice at 10 dpn. (*B*) *Alpl* ^−/−^ mice injected with AAV8‐TNAP‐D_10_ within 5 dpn were not significantly smaller than WT mice at 15 dpn. Statistical analysis was performed by one‐way ANOVA followed by Turkey's multiple comparison test.

**Supplemental Fig. S2.** Histochemical staining of the liver reveals no ALP activity in the branches of hepatic artery of AAV8‐TNAP‐D_10_‐treated *Alpl* ^−/−^ mice, which is apparent in WT mice (black arrow).

**Supplemental Fig. S3.** Von Kossa staining shows no ectopic calcifications in the aorta, coronary arteries, brain, or kidney in WT and AAV8‐TNAP‐D_10_‐treated *Alpl* ^−/−^ mice at 70 dpn.

**Supplemental Fig. S4.** Safranin O staining of the decalcified tibias shows an abnormal distribution of chondrocytes in secondary ossification centers in AAV8‐TNAP‐D_10_‐treated *Alpl* ^−/−^ mice (black arrows). Slides were scanned by Aperio AT2 system to capture images of the entire tibia. The WT and *Alpl* ^−/−^ images shown in the upper panel were the same as those shown in Fig. 4*C* (observed with microscopy).

**Supplemental Fig. S5.** Hypomineralization and impaired periodontium in untreated *Alpl* ^*−/−*^ teeth compared with WT mice. (*A*) H&E staining of the first mandibular molar showing hypomineralized molar roots (upper panel, red arrow). The lower panel shows hypoplasia of acellular cementum (*) and loss of periodontal attachment. (*B*) ImageJ color map of H&E‐stained images showing abnormal PDL cells in *Alpl* ^*−/−*^ mice versus WT mice. (*C*) Bar graph of cell counts in the boxed region showing fewer PDL cells, although not significant, in *Alpl* ^*−/−*^ mice compared with WT mice. Statistical analysis was performed by Student's *t* test.

## Data Availability

The data that support the findings of this study are available from the corresponding author upon reasonable request.
