# Supplementary figures and images for: Gene Therapy Using Adeno‐Associated Virus Serotype 8 Encoding TNAP‐D10 Improves the Skeletal and Dentoalveolar Phenotypes in Alpl −/− Mice
Source: J Bone Miner Res. 2021 Jun 15;36(9):1835–49. doi: 10.1002/jbmr.4382 (PMC8446309; doi:10.1002/jbmr.4382)

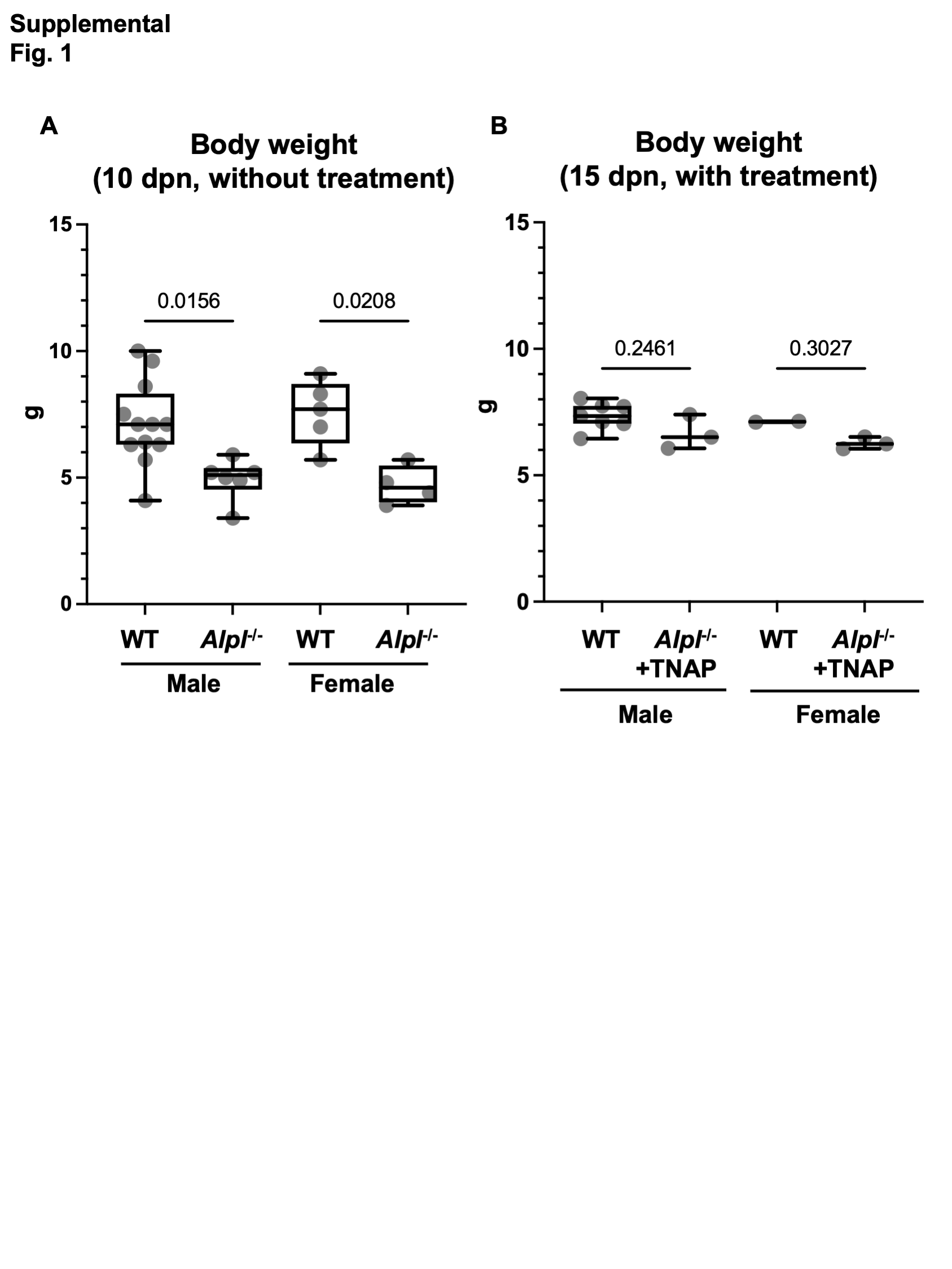

Supplement: Supplementary file 1 — Supplemental Fig. S1. (A) Untreated Alpl −/− mice were significantly smaller than WT mice at 10 dpn. (B) Alpl −/− mice injected with AAV8‐TNAP‐D10 within 5 dpn were not significantly smaller than WT mice at 15 dpn. Statistical analysis was performed by one‐way ANOVA followed by Turkey's multiple comparison test. [file JBMR-36-1835-s002.tiff]

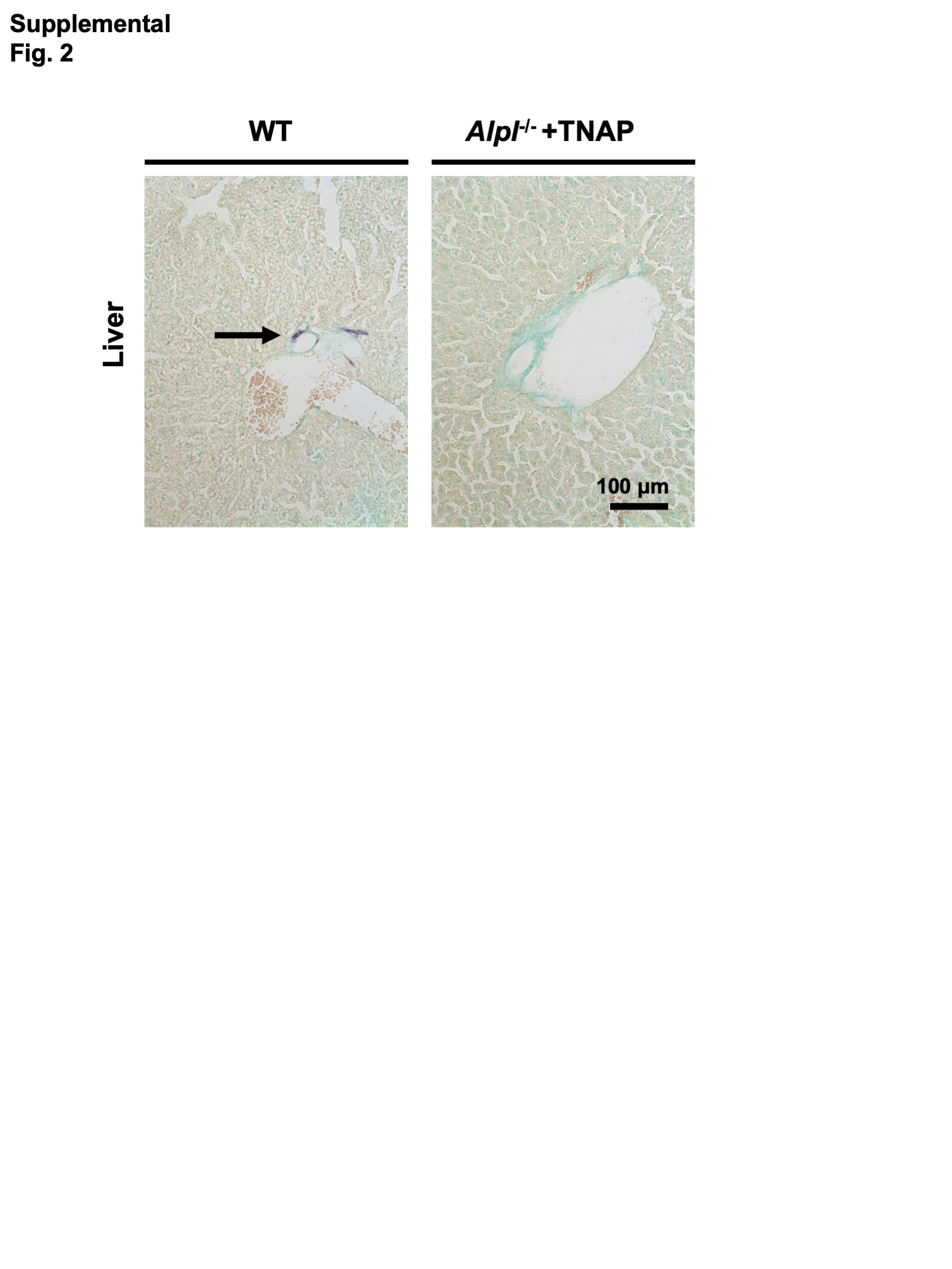

Supplement: Supplementary file 2 — Supplemental Fig. S2. Histochemical staining of the liver reveals no ALP activity in the branches of hepatic artery of AAV8‐TNAP‐D10‐treated Alpl −/− mice, which is apparent in WT mice (black arrow). [file JBMR-36-1835-s005.tiff]

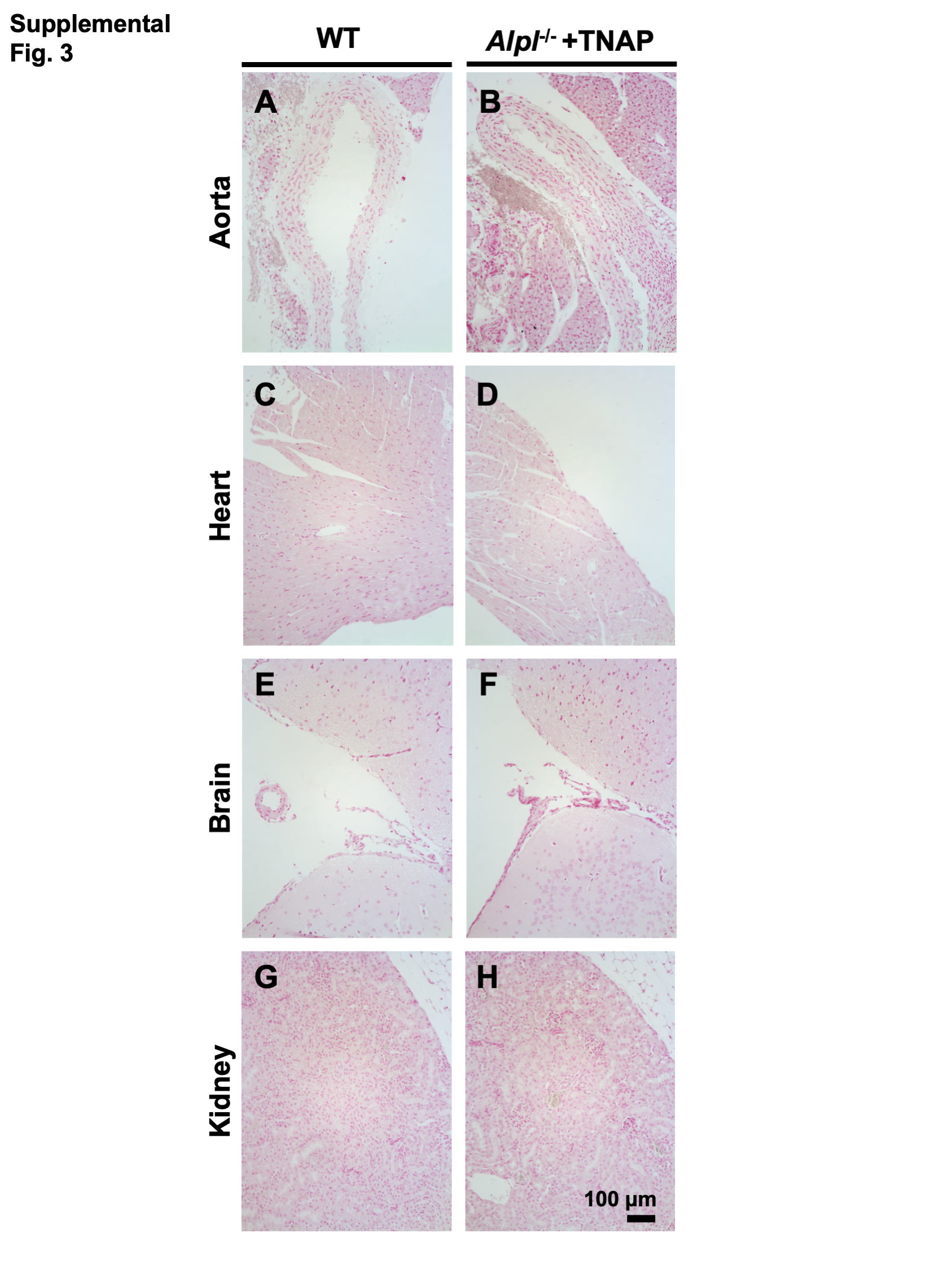

Supplement: Supplementary file 3 — Supplemental Fig. S3. Von Kossa staining shows no ectopic calcifications in the aorta, coronary arteries, brain, or kidney in WT and AAV8‐TNAP‐D10‐treated Alpl −/− mice at 70 dpn. [file JBMR-36-1835-s001.tiff]

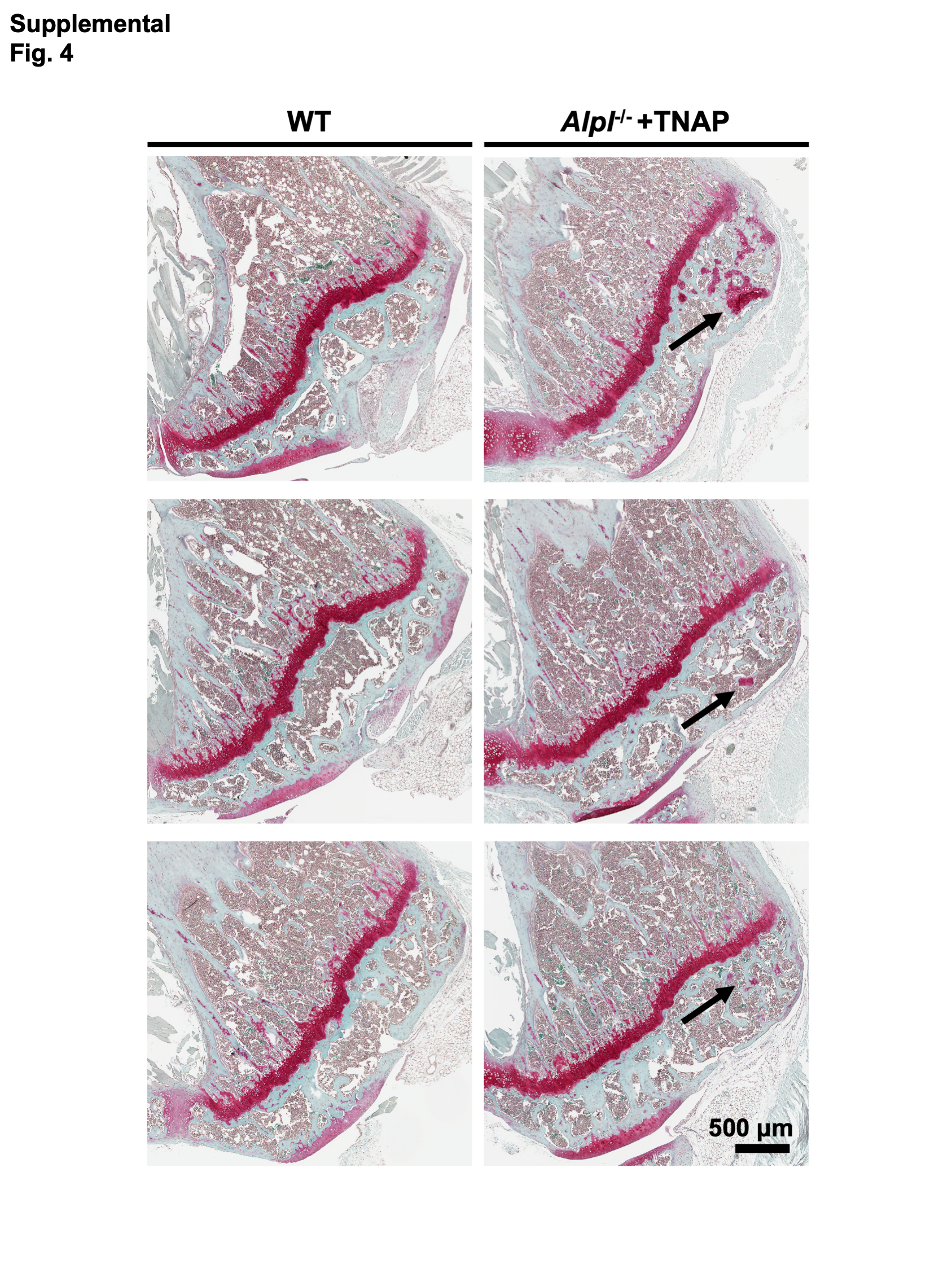

Supplement: Supplementary file 4 — Supplemental Fig. S4. Safranin O staining of the decalcified tibias shows an abnormal distribution of chondrocytes in secondary ossification centers in AAV8‐TNAP‐D10‐treated Alpl −/− mice (black arrows). Slides were scanned by Aperio AT2 system to capture images of the entire tibia. The WT and Alpl −/− images shown in the upper panel were the same as those shown in Fig. 4C (observed with microscopy). [file JBMR-36-1835-s004.tiff]

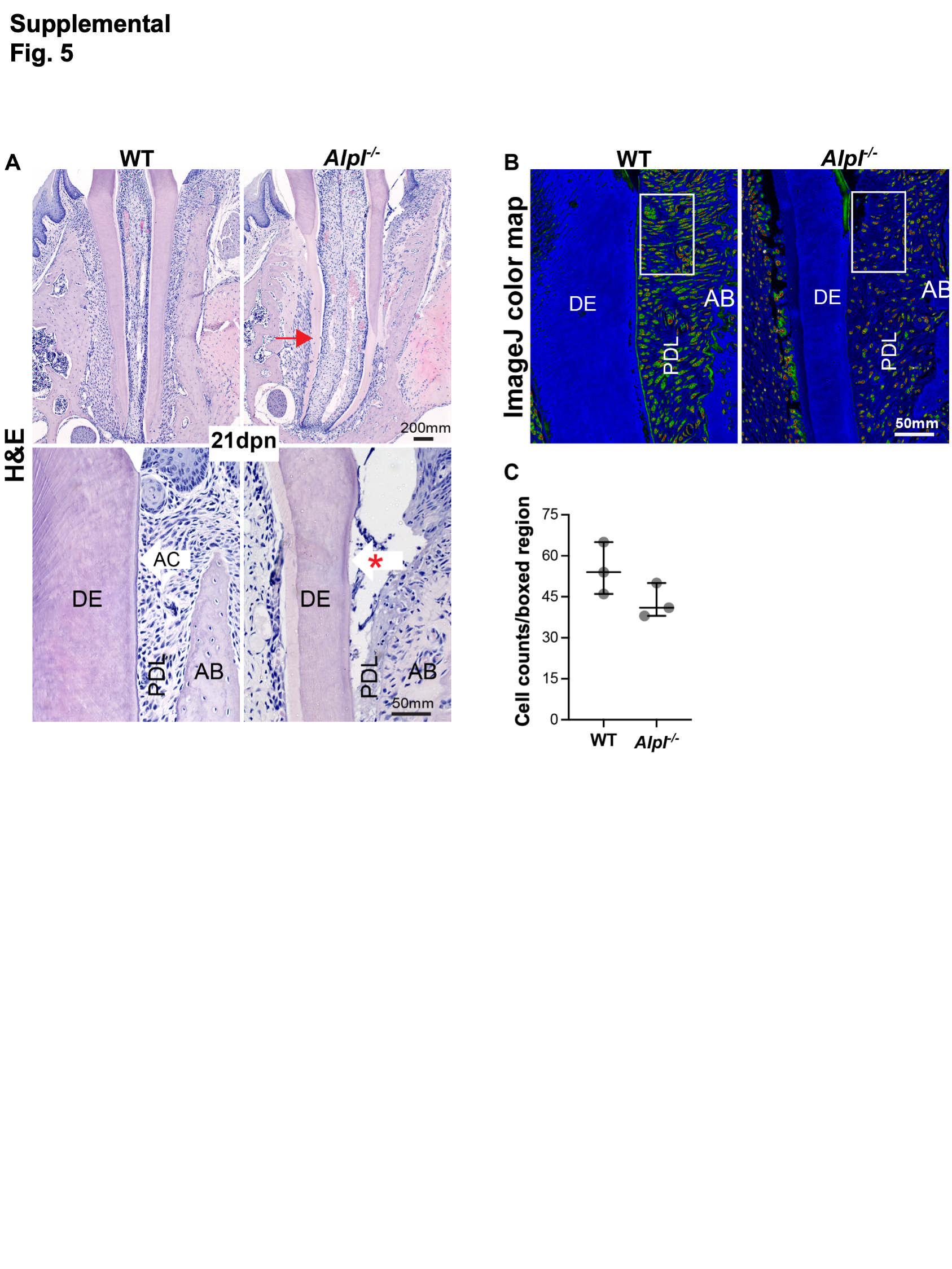

Supplement: Supplementary file 5 — Supplemental Fig. S5. Hypomineralization and impaired periodontium in untreated Alpl −/− teeth compared with WT mice. (A) H&E staining of the first mandibular molar showing hypomineralized molar roots (upper panel, red arrow). The lower panel shows hypoplasia of acellular cementum (*) and loss of periodontal attachment. (B) ImageJ color map of H&E‐stained images showing abnormal PDL cells in Alpl −/− mice versus WT mice. (C) Bar graph of cell counts in the boxed region showing fewer PDL cells, although not significant, in Alpl −/− mice compared with WT mice. Statistical analysis was performed by Student's t test. [file JBMR-36-1835-s003.tiff]
